# Reducing Sugar Intake in South Africa: Learnings from A Multilevel Policy Analysis on Diet and Noncommunicable Disease Prevention

**DOI:** 10.3390/ijerph191811828

**Published:** 2022-09-19

**Authors:** Nicole McCreedy, Maylene Shung-King, Amy Weimann, Lambed Tatah, Clarisse Mapa-Tassou, Trish Muzenda, Ishtar Govia, Vincent Were, Tolu Oni

**Affiliations:** 1 Health Policy and Systems Division, School of Public Health and Family Medicine, University of Cape Town, Cape Town 7925, South Africa; 2 Research Initiative for Cities Health and Equity (RICHE), Division of Public Health Medicine, School of Public Health and Family Medicine, University of Cape Town, Cape Town 7925, South Africa; 3 African Centre for Cities, University of Cape Town, Cape Town 7701, South Africa; 4 Health of Populations in Transition Research Group (HoPiT), University of Yaoundé I, Yaoundé P.O. Box 812, Cameroon; 5 Global Diet and Physical Activity Research Group, Medical Research Council Epidemiology Unit, University of Cambridge, Cambridge CB2 0QQ, UK; 6 Department of Public Health, Faculty of Medicine and Pharmaceutical Sciences, University of Dschang, Dschang P.O. Box 96, Cameroon; 7 Caribbean Institute for Health Research, The University of the West Indies, Kingston 7, Jamaica; 8 Center for Global Health Research, Kenya Medical Research Institute (KEMRI), Kisumu 40100, Kenya

**Keywords:** noncommunicable diseases, NCDs, sugar, sugar-sweetened beverages, SSBs, South Africa

## Abstract

High sugar intake contributes to diet-related excess weight and obesity and is a key determinant for noncommunicable diseases (NCDs) in low- and middle-income countries (LMICs). The World Health Organization (WHO) gives specific advice on limiting sugar intake in adults and children. Yet, to what extent have policy ideas on sugar intake reduction originating at the global level found expression at lower levels of policymaking? A systematic policy document analysis identified policies issued at the African regional, South African national and Western Cape provincial levels between 2000 and 2020 using search terms related to sugar, sugar-sweetened beverages (SSBs), and NCDs. Forty-eight policy documents were included in the review, most were global and national policies and thus the focus of analysis. A policy transfer conceptual framework was applied. Global recommendations for effectively tackling unhealthy diets and NCDs advise implementing a mix of cost-effective policy options that employ a multisectoral approach. South African country-level policy action has followed the explicit global guidance, and ideas on reducing sugar intake have found expression in sectors outside of health, to a limited extent. As proposed in this paper, with the adoption of the SSB health tax and other policy measures, South Africa’s experience offers several learnings for other LMICs.

## 1. Introduction

Excess sugar intake, from sugar added to food products and especially sugar-sweetened beverages (SSBs), is considered a major contributor to excess weight and obesity in adults and children [1,2]. Excess weight and obesity are associated with the steady rise of non-communicable diseases (NCDs) such as cardiovascular disease (CVD), type 2 diabetes, and various cancers. In 2019, NCDs were estimated to have caused 40.8 million deaths (73.6% of all deaths) globally [3]. Around three-quarters of these deaths occur in low-and middle-income countries (LMICs) such as South Africa, where chronic and infectious diseases coexist [3,4]. In the African region, NCDs are responsible for 2.9 million deaths (37.1% of all deaths) and health systems already under strain are struggling to address the growing plight [3,5]. In 2019, in South Africa, 254,700 (51.3% of all deaths) were due to NCDs [3].

An enabling factor is that in recent decades globally, and in LMICs, people’s eating habits have changed [6]. Unhealthy diets high in calories, sugar, salt and fats have contributed to excess weight gain in local populations putting people at higher risk for NCDs. Excess weight and obesity are now commonplace [7]. In South Africa, half the adult population are either overweight (23.6%) or obese (26.2%) [8]. Several factors can influence a person’s food choices and dietary habits [9,10]. These vary from individual preference, knowledge or behaviors to household circumstances, cultural norms or characteristics within their local neighborhood or school environment. 

High sugar consumption is also associated with dental caries—another preventable NCD—and children who consume sugary drinks are especially vulnerable [11,12]. In 2015, to curb excess sugar intake at a population level, the World Health Organization (WHO) released guidelines on sugar intake for adults and children [11]. The guidelines recommended that in both adults and children, the intake of free sugars be reduced to less than 10% of total energy intake. South Africans are estimated to consume up to 24 teaspoons of sugar per day—double the daily WHO recommendation [13]. Seeking to address the situation in 2018, the South African government introduced the Health Promotion Levy (HPL), a tax on sugary drinks to reduce the population’s sugar consumption [14].

South Africa produces its own sugar and has a large SSB industry–making the crop economically important [15]. The country also has high unemployment and, as a result, the HPL encountered opposition from both the business sector and trade unions, which led to delays in instituting the tax [14,16]. Nonetheless, South Africa is among the few LMICs, and (the only African country after Mauritius, until Nigeria joined recently) to have implemented a tax on sugar products for health reasons [17]. The HPL is one of several measures the government has employed to tackle the multifaceted problem of NCDs and their risk factors [18]. Therefore, as other countries in the region eventually seek to explore policy options to address unhealthy diets, it would help to understand how global policy directives and recommendations on sugar intake to prevent and control NCDs have found expression in the South African context.

Health policy analysis (HPA) in LMICs is a relatively underdeveloped area of research [19]. This study offers an enquiry into a theory of policy development that describes a process or set of processes in which policies from one governance setting inform those of another, commonly known as policy transfer [20,21,22,23]. Specifically, this study is a retrospective policy document analysis that explores whether and how policy ideas on sugar intake reduction held in the global system of governance have found expression in Africa, particularly in South African policies. 

The specific interest of this study is on the transfer of ideas from a global to national level where strategic and population-level decisions related to health and wellbeing are made. However, as globally-dictated policy is not always appropriate or feasible in a local country context—for example countries may find different, or innovative ways to address challenges that require a global response—examining the transfer of ideas may provide useful bi-directional learnings. In short, lessons coming from the bottom up may contain applicable insights that are just as important for policy formation as directives coming from the top down. Expressions of transfer can also occur between institutions or laterally between countries [24,25,26].

The policy transfer conceptual framework chosen for this analysis provides a structured approach for examining the extent to which global dictates have influenced country-level policy action for South Africa [20,21]. Specifically, we explore policy transfer in the context of NCDs and the much-contested area of sugar reduction as a possible intervention to tackle excess weight and obesity. To the best of our knowledge, the conceptual framework proposed for this study has not been applied within the context of health policy analysis and LMICs before. Nor has policy transfer been examined in relation to diet and the prevention of NCDs or the other specific subjects of interest in this study. 

## 2. Conceptual Framework 

The Dolowitz and Marsh (1996 and 2000) framework chosen for this research is a well-regarded heuristic model that covers a wide range of policymaking activities. The various scopes of policy transfer are organized into a framework structured around six empirical questions, namely; “Why do actors engage in policy transfer?”; “Who is involved in the policy transfer process?”; “What elements of policy are transferred?”; “From where are lessons drawn?”; “What are the different degrees of transfer?”; “What restricts or facilitates the policy transfer process?” and “How successful is the policy that was transferred?” [20,21]. 

The framework provides a useful explanatory tool that helps shed light on the variables involved in the policy transfer process and presents one perspective on policy change (Supplementary Material, Figure S1). Moreover, it places a strong emphasis on the national context and therefore lends itself, with some adaptation, to our purpose of exploring the uptake of policy ideas emanating from global governance at the South African national and sub-national levels [20,21]. Although not explored in this study, the multidirectional flow of policy ideas actions is recognized and presents an opportunity for further investigation at a later stage. Furthermore, while implementation or practice is an important aspect of policy, it was not examined in this study since our primary source of analysis was from formal written policy documents.

## 3. Materials and Methods

This multilevel policy document analysis was undertaken as part of global and regional research by the Global Diet and Activity Research Group and Network (GDAR), funded through the National Institute for Health Research Global Health Research initiative. The GDAR project methodology is published in the Protocol for a Multi-Level Policy Analysis of NCD Determinants of Diet and Physical Activity: Implications for LMICS in Africa and the Caribbean [10]. The protocol provides further details of the methods used in this paper in relation to the search strategy, document selection criteria, data extraction and coding of documents. 

The search terms for this study included “noncommunicable disease” or “chronic disease”; “non-infectious disease” (including non-hyphenated variations on spelling), as well as “diet”, “nutrition” and “sugar (s)”; “glucose” and “sugar-sweetened”. Only official policy documents where sugar was mentioned in relation to the prevention and control of NCDs were included in this study. Supplementary searches were conducted at the global level to identify sugar-specific policies and documents collected during the first phase of data collection done as part of the GDAR scope of work [10]. The document selection criteria were considered broad enough to assess official health policy directives and recommendations at the global level while still providing the depth required for multilevel analysis into policy transfer at the lower levels of governance.

NVivo 12 software was used to code and thematically analyze the data. A content analysis was the analytical approach applied, involving a thematic analysis of the content that employed both deductive and inductive themes. The Dolowitz and Marsh (2000) policy transfer framework was used to establish the deductive themes, as well as guiding the detailed approach to content analysis. Each category of this framework is described in a codebook (Supplementary Material, Table S1), which can potentially be used as a resource by other health policy analysts in LMIC contexts [20,21]. 

## 4. Results

**Figure 1:**
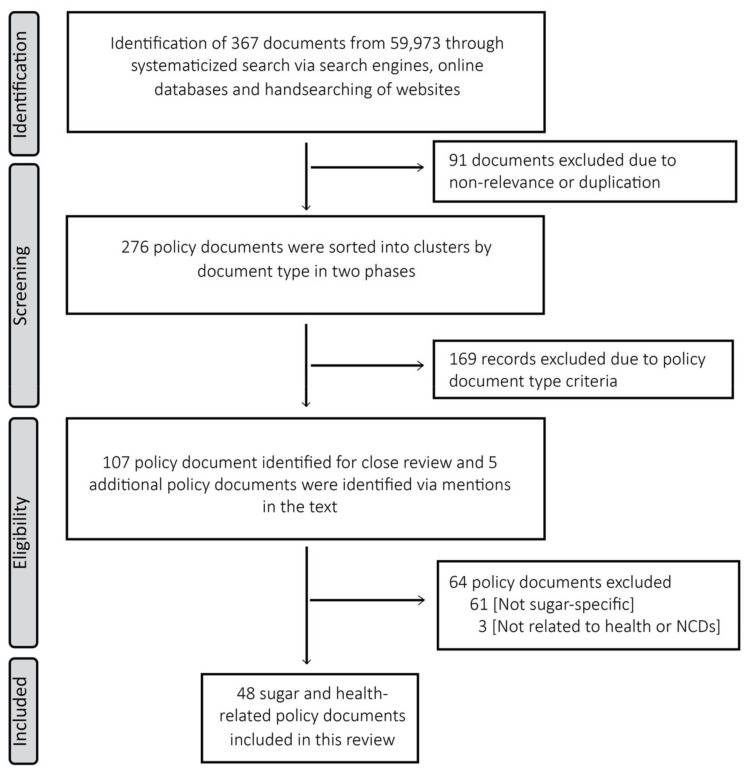
Article selection flow chart.

A total of 48 documents were finally included in this study (Figure 1), representing a selection of global, African, South African and Western Cape regional policies (Supplementary Material, Table S2). At the different lower levels of governance, it appears that, depending on the context, the global policy directive most strongly assumed in relation to sugar intake and NCDs depends on the health priority. In other words, at an African Regional level the two of the three policy documents found relate to oral health, the most recent having an explicit focus on NCDs, whereas at the national level the key priority appears to be the prevention and control of excess weight and obesity [27,28,29,30].

Application of the conceptual framework for the policy document analysis provided insight into several aspects of transfer (Table 1), which are subsequently expanded upon in the results section. Using the structured approach, the framework enabled lessons to be drawn for the discussion.

### 4.1. Who Is Involved in the Policy Transfer Process and Why Engage in Transfer?

#### 4.1.1. International Organizations: The WHO/FAO and Codex

##### The FAO

The characterization of unhealthy diets as a contributing factor to the global NCD burden strongly emerges with the joint WHO/FAO Expert Consultation on Diet, Nutrition and the Prevention of Chronic Disease held in 2002 and the report detailing their recommendations [31]. In the FAO’s 2004 follow-up report that was put forward to the Committee on Agriculture (COAG), the FAO critically highlights that the overconsumption of sugar is associated with NCD development and provides the voluntary nutrient-specific goal for reducing sugar intake at a population level. The group of experts representing the WHO/FAO recommend limiting the intake of free sugars to 10% or less of total energy per day [31]. 

##### The WHO

The WHO’s methods for influencing policy range from organizing epistemic policy communities and global high-level and regional meetings; to producing technical briefs and establishing the United Nations Interagency Taskforce on NCDs (UNIATF). The WHO has also created know-how-to tools for putting the NCD best-buys into action, and created online data- and knowledge-sharing portals, for example the WHO e-Library of Evidence for Nutrition Actions (eLENA) [32]. In fact, the current global focus on reducing sugar consumption reflects the WHO’s overall influence on the international health agenda. Amongst other things, the WHO has helped to prioritize the issue of high-sugar content in SSBs and the ways in which the over-consumption of SSBs can lead to negative health consequences [11]. In recent years, the WHO has deliberately educated policymakers on how to implement health taxes, including guidance on reducing SSB consumption [33,34]. 

##### The Codex

The Codex, an international organization run jointly by the FAO and the WHO, has been instrumental in mobilizing the agenda on improving consumer awareness of the sugar content in foods through food labelling and nutrition claims related to sugar [35,36]. The global regulatory environment on nutrition labelling and health claims on food relies on a set of voluntary guidelines drawn up by the Codex that subsequently inform national regulations [37]. In addition, the Codex documents in this review offered insight into the inner workings of the policy process, particularly the contestation between actors who enact their power during policymaking, as the process brings government and food industry representatives into a space for negotiation [36,38]. 

Since WHO member states were urged through resolution WHA56.23 (adopted in 2003) to make full use of the Codex “*throughout the food chain, including assistance with making healthy food choices regarding nutrition and diet*”, South Africa has aimed to follow its standards and guidelines [35]. These standards and guidelines are expressed in South Africa’s national regulations relating to the labelling and advertising of foods [32,36,39,40]. South Africa is also a member of the Codex Alimentarius Commission (CAC), which meets annually, and there is also a Regional Coordinating Committee for the Africa region that holds regular meetings for members to contribute on key topics related to food safety and food labelling [41]. Participation in these decision-making structures provides a channel for countries to have a say in policy formation and be influenced in their thinking by other members and global ideas. Participation by members states in Africa is reportedly variable—South Africa being one of the more regular meeting participants [41]. 

#### 4.1.2. National Government

The global policy documents clearly stipulate that national governments have “stewardship” for acting on global strategies and plans to address NCDs; this involves choosing which policy programs to implement, depending on local context and policies, and proactively leading multisectoral arrangements [35]. However, according to reports by UNIATF, LMICs have faced challenges to employing more technical policies such as health taxes, due to a lack of “*analytical, legal and tax administrative*” skills capacity and policy expertise [42].

#### 4.1.3. The Private Sector 

One of the key sources of tension in the policy transfer discussion is between the private sector and government. The private sector as manufacturers and retailers of food products play an important role in the formulation of processed foods and the amount of sugar these types of foods contain. Multinational actors from the food and beverage industry have a major presence in LMICs, including in South Africa, where they have introduced highly-developed supply management systems, centralized purchasing and distribution, product differentiation, and specialized marketing techniques, often targeting children [43]. These entities and their representative associations are also known to oppose government authorities through effective lobbying [36,44]. 

The WHO Global Action Plan on the Prevention and Control of NCDs (2013–2020) identified fiscal measures as a mechanism for creating a supportive environment for reducing NCD risk factors and called on governments to lead the development of a national policy framework to promote health and address the risk factors in question [45]. However, reducing sugar consumption through the effective taxation of SSBs was only introduced as a best-buy in the Updated Appendix 3 to the WHO Global Action Plan (2013–2020), endorsed in 2017 at the World Health Assembly (WHA) [46]. The step was taken as sugary drinks have increasingly come under the spotlight for their role in influencing weight gain in adults and in children, and as more evidence on the causal relationship has become available [1,32]. The UNIATF, as well as the WHO Regional Office for Africa, have also cautioned that private sector interference and conflict are a problem at the country-level [42,47]. Specific mention is made of private sector interference at country-level to stall cost-effective measures, for example instituting excise taxes on SSBs (Table 2). 

### 4.2. From Where Are Policy Directives Drawn?

In 2004, the WHO Global Strategy on Diet, Physical Activity, and Health outlined recommendations for the promotion of healthy diets and the restriction of unhealthy ones in its principal strategic document. The subsequent proposals took shape through action plans to prevent and control NCDs, and were rolled out in 2008 and 2013 [45,49]. The WHO directives issued in 2004 provide a reliable representation of policy options at the global level and have demonstrably found expression in the South African context (Table 3). 

#### 4.2.1. Global Level Key Policy Ideas 

In this policy analysis, several policy ideas were identified at the global level. These ideas can be broadly structured as the ‘problem frame’; the ‘solution frame’ (multisectoral and cost-effective approaches); and as a set of prescribed actions in global policies aimed at national governments.

##### Recognition at a Global Level That Unhealthy Diets and High Sugar Intake Are Related to NCD Burden 

By the early 2000′s, enough epidemiological evidence on NCDs and the nutrition transition has been collected at the national level to draw global attention to the role of unhealthy diets as a risk factor for NCDs [31]. As mentioned, the accumulated evidence and risks were presented by the WHO/FAO around that time, accompanied by the rationale that reducing excess sugar consumption would have both a nutritional benefit and help individuals to avoid dental caries and/or help to mitigate the chances of developing excess weight or obesity (a significant risk factor for NCDs). As specified in the WHO/FAO recommendation:

*“Limiting high intakes of free sugars, which provide energy without specific nutrients and increase the risk of unhealthy weight gain, improves the nutritional quality of diets and decreases the risk of dental decay”.*

[31]

##### Recognition of the Added Vulnerabilities of Women and Children, Especially in An Urban Context

Since the late 2000s, there has been an increased focus on the particular impact of unhealthy diets on women and children [50]. In 2010, the issue of marketing and advertising foods and non-alcoholic beverages to children was formally recognized as a priority on the global health agenda with the WHA63.14 resolution, which gave a clear set of recommendations for member states to follow [51,52]:

*“…children should maintain a healthy weight and consume foods that are low in saturated fat, trans-fatty acids, free sugars, or salt in order to reduce future risk of noncommunicable diseases”.*

Notably, the evidence for a direct causal link between sugar or SSBs and NCDs only materialized a few years later and was only made mainstream in 2015, based on the WHO Nutrition Guidance Expert Advisory Group (NUGAG)’s evidence-review of the scientific rationale between high-sugar intake, obesity and dental caries (see the WHO Guideline: Sugars Intake for Adults and Children) [53]. The Guideline’s daily recommended sugar intake remained the same as when FAO/WHO experts first made a recommendation in 2002, but the 2015 document makes clear that this recommendation applies for both adults and children [11]. Crucially, according to this guideline, there is an explicit link between the consumption of sugary drinks and excess weight gain in children [32]. As the document states:

*“…children with the highest intakes of sugar-sweetened beverages had a greater likelihood of being overweight or obese than children with the lowest intakes”*.

##### A Multi-Sectoral Approach and Mix of Policy Options Is Needed to Address the Underlying Structural Determinants of NCDs

From the global documents under review, two recommended approaches/responses strongly emerge for countries to follow in addressing the determinants of NCDs, namely, a multisectoral and a cost-effective approach. Firstly, with regard to the multisectoral approach, the documents acknowledge how wider structural drivers such as globalization, industrialization, urbanization and trade have impacted the food chain; increased the production and consumption of processed foods high in fats, salt and refined sugar; and ultimately contributed to weight gain and increasing levels of NCD in the global population [31,50]. However, this is not to say that the causes of NCDs are only structural. Indeed, due to the complex combination of factors influencing people’s food choices and health, a singular approach to combating the global burden of NCDs cannot suffice, and governments are therefore recommended to employ a “*mix of actions in accordance with their national capabilities and epidemiological profile*” [35]. This then, is the significance of the multisectoral approach, and it is no surprise to see it repeatedly and explicitly emphasized in the global policy documents in this analysis.

##### A Set of Cost-Effective Interventions or “Best-Buys” Are Available for LMICs 

LMICs have competing healthcare priorities and while the long-term health and economic impacts of NCDs can be high, prioritizing these slow-developing chronic health conditions can be a challenge. It is also the case, however, that the common behavioral risk factors for NCDs are largely avoidable and can be addressed through “simple interventions” that are cost-effective to implement [54]. The second response of global organizations is, therefore, to promote a set of cost-effective interventions or a “best-buys” menu that provides potentially implementable solutions that maximize returns on investment in health at the population-level to help support government financial constraints.

In 2007, the WHO Director-General was requested “to disseminate to Member States, in a timely and consistent manner, information on cost-effective, core interventions aimed at preventing and controlling NCDs” [54]. By taking this course of action, the Director-General intentionally instigates a process for facilitating the transfer of policy ideas around what the WHO proposed as cost-effective interventions for implementation nationally, some of which would require a multisectoral approach for favorable implementation [55]. The UNGA report on the Prevention and Control of NCDs (A/66/83) states that:

*“Best buys to reduce major risk factors for noncommunicable diseases include:*

*Reducing salt and sugar content in packaged and prepared foods and drinks”.*

[50]

To help LMICs with combating population-level NCDs, the policy options identified in the 2004 WHO Global Strategy were subsequently organized in the WHO Global Action Plan (2013–2020) in terms of their cost-effectiveness or the “road-map” member states are suggested to follow [45]. Unlike “reducing salt intake”, limiting sugar intake was not explicitly listed among the nine time-bound targets or in the table of cost-effective interventions identified in the plan, which followed on from the commitment made by nation states in the UN Political Declaration on the Prevention and Control of NCDs (resolution A/RES/66/2) [56]. This omission potentially hampers policy action in this area. However, governments are advised in the plan to develop guidelines, recommendations or policy measures that do encourage the reduction in sugar intake in foods and non-alcoholic beverages [45].

#### 4.2.2. Expression of Global Ideas in South African Policies

As a member of the WHO and a signatory of various high-level declarations, South Africa has made a commitment to prevent and control NCDs. These commitments are voluntarily expressed in the South African Declaration on the Prevention and Control of NCDs signed at a ministerial level, and in strategic documents issued by the Department of Health [28,57,58]. The idea that excess sugar consumption plays a critical factor in NCD development is articulated in South African policy statements [28]. However, there is some variation in the way that policies around sugar consumption find representation in South Africa as compared to global entities such as the WHO/FAO. 

##### South African Recommendation and Definition of “Added Sugars”

Concomitant with the global directive on healthy diets and limiting sugar intake, the 2007 South African regulations relating to food labelling and advertising added an annexure (No. R642) to clarify which types of ingredients, such as sugar or manufactured products with added sugar, constitute an unhealthy diet [59]. South African regulators and the WHO use slightly different terminology and definitions. “Added sugars” is used consistently by South African regulators despite the WHO preference for “free sugars”, showing some independence from global terminology [40]. In the South African policymakers’ definition, there is also greater specificity possibly to guide implementers (see Table 4). Though it is unclear whether there is any practical implication of this (aside from possibly using terminology that is more comprehensible in the local context) [53].

In keeping with the suggestion from the WHO/FAO Expert report, the FBDG-SA 2013 chapter on sugar and health advises that “*an intake of added sugar of 10% of dietary energy is an acceptable upper limit*” [53]. Although not specified in the global recommendation, the FBDG-SA 2013 suggests a lower limit for sugar intake is set at 6%. This is, however, qualified as applying to at-risk groups, such as individuals who are overweight or have prediabetes [53].

##### Sugary Drinks Contribute to Excess Weight and Obesity in Sub-Populations 

Similar to their global counterparts, South African policymakers in the FBDG-SA 2013 make several mentions of excess weight and obesity, particularly as an “acute” problem affecting women and urban residents [53]. Children are also identified as being at risk of excess weight [53]. This is significant as it recognizes the intersecting effect of urbanization and the vulnerability of key subpopulations simultaneously. This recognition of intersecting factors is similarly emphasized in the (national) South African Strategy on the Prevention and Control of Obesity (2015–2020), and the (provincial) Western Cape Government Household Food and Nutrition Security (Strategic Framework) from 2016. In the latter, it is specifically emphasized that that excess weight and obesity are on the rise and that urban youth are especially vulnerable, “*with the highest fat and sugar scores found in the youngest age groups, in formal urban areas in those provinces that were largely urbanised*” [29]. The recognition that disparate factors such as age, gender, and geography can overlap to produce particularly vulnerable populations possibly suggests the forward-thinking nature of subnational policymaking but cannot be categorically stated as such. 

##### National Policy Documents Endorse a Multi-Sectoral Approach and Apply Cost-Effective Approach

The national application of the “best-buys” concept is also reflected within the national NCD Plan, with a chapter entitled “*Cost-effective interventions for addressing NCDs*”, where interventions are stratified according to their cost-effectiveness [58]. In the Strategic Plan for the Prevention and Control of NCDs (2013–2017), the cost in rand per head is calculated and compared across a range of intervention measures [58], with fiscal measures proving to be the most cost-effective of those on the list. These findings are also presented later in the Strategy on the Prevention and Control of Obesity (2015–2020) where taxes on sugary foods were characterized as “*potential cost-effective strategies for addressing diet and obesity*” [28]. The report goes on to emphasize that “*a multiple-intervention approach is essential to see substantially larger health gains”* in contrast to interventions at a purely individual level. In countries where a multiple-intervention approach has been implemented to address obesity, the report goes on, cost savings have been realized [28]. In South Africa, policymakers have assimilated this approach and taken the initiative by assessing policy options through a health-economics lens. 

##### A Mix of Policy Options Applied in the South African Context 

It is evident from the policy documents, that a set of prescribed actions are available as options for the government to choose from to address unhealthy diets and encourage healthier ones. For our purposes, these documents were organized into four cross-sectoral clusters aimed at restricting the consumption of unhealthy foods and sugar. The four clusters were: food-based dietary guidelines; marketing and advertising of foods and non-alcoholic beverages (especially to children); food labelling; and fiscal policies—specifically relating to taxes on SSBs. These South African policy documents are identified above in Table 3.

##### Gaps Identified in Policies Targeting Sugar Intake

The decision to organize the South African policy documents into four cross-sectoral clusters was informed both by global strategy recommendations and the initial policy document review and coding of documents. The policy stream approach helped to visually reveal gaps where policies appeared absent or thin at the national and sub-national levels. At the global level, sugar intake policies impacting health and agriculture were most common, whereas at the national level sugar intake policies impacting education and finance were also present. 

One policy gap that was identified at the national level for South Africa was that the country does not have regulations concerning the marketing and advertising of foods and non-alcoholic beverages to children. The South African Department of Health regulations relating to the labelling and advertising of foodstuffs (No. R642) of 2007 did include the following clause that prohibits the marketing, advertising, and promotion of certain food items to children younger than 16:

*“a foodstuff not regarded essential as part of a healthy diet and healthy lifestyle, as listed in Annexure 6 …shall not advertise or promote in any manner, in any school tuck shop or on any school or pre-school premises”.*

[60]

However, despite the mention of children in the 2007 regulation (No. R642), the regulations relating to the labelling and advertising of foodstuffs issued in 2010 (No. R146) makes no such mention. This was remedied in 2014 with a new regulation (No. R429) (gazette but not passed into law), which introduced a section on the commercial marketing and advertising of foods and non-alcoholic beverages to children stating “*no food should be marketed to children unless it complies with Guideline 14*” [40]. Unfortunately, Guideline 14 was not located as a standalone policy document in this review, as it was not retrievable from the Department of Health website [61]. The absence of more stringent local regulation is conspicuous given the length of time that this issue has sat on the global policy agenda and the attention that has since been given by the WHO to excess weight and obesity in children.

### 4.3. Degree of Policy Transfer

Drawing on Rose (1993), Dolowitz and Marsh highlight the following categories for determining the degree of policy transfer that occurs between entities: copying from another context, emulation, hybridization and synthesis (which they combine as mixtures), and inspiration [20,62]. Copying and emulation were the two forms identified in this review.

#### 4.3.1. Copying 

Copying refers to the use of existing policy as is, without alteration and may use the exact wording [63]. In South Africa, examples of policy copying are most evident within the regulations present on food labelling and in the advertising of foodstuffs and non-alcoholic drinks. These product food labels communicate to consumers the nutrition content and health benefits of foods, allowing them to assess whether a product has a high-sugar content and whether to avoid it. Food labelling is also important when a tax is applied, such as to sugar in SSBs, as a source of information on the number of grams of the ingredient [64]. In 2014, South Africa amended the previous regulations relating to the labelling and advertising of foods (No. R146) to include important proposed changes [39,40]. The amendment (No. R429) included, among other things, introducing mandatory nutritional labelling, reducing disease-risk claims to include the mention of NCDs, and the inclusion of non-addition claims for sugar(s) [40]. As shown in Table 5, the wording used by South African authorities often closely resembles the language used by the major global health organizations [40,65]. These regulations show the South African government’s progressive commitment to making consumers aware of their sugar intake and the influential role of the Codex in the transfer of standards.

#### 4.3.2. Emulation

In this policy analysis, it is arguably emulation, which assumes a “*standard basis starting point for best policy but allows for adjustment to suit varying needs of the adopter*” that is the most common approach [63]. In terms of emulation, this is best exemplified by the South African sugar tax. Countries have introduced taxes on SSBs for a variety of reasons—including to achieve positive health impacts or generate revenue. Although the UNIATF proposes this as mechanism in order to ensure self-financing of national responses to NCDs, it has also noted that countries can have insufficient capacity to increase domestic taxes on health-harming products [42]. 

The case for emulation here is demonstrated by the government’s rationale in determining the tax to be a cost-effective approach, as outlined in strategic policy documents [58]. In 2013, South African policymakers made the proposal before WHO included taxation of SSBs to its list of cost-effective interventions in 2017 [46]. However, both the ideas of reducing sugar intake for the prevention and management of NCDs and the use of cost-effective interventions were promoted by the WHO. South Africa has also in the past, until 2002, had a tax on soft drinks, for revenue reasons, and has experience in this area [66]. South African policymakers were thus capable to assimilate these “best policy” ideas—and, somewhat unusually, they have been explicit about using this tax for the purposes of promoting population health. Indeed, the official name for the sugar tax is the Health Promotion Levy (HPL), an appellation deliberately intended to reflect the policy intention [64]. The HPL tax is leveraged on beverages where the sugar content exceeds the threshold of 4 g per 100 milliliters. This applies also to cocoa powders, malt drinks, syrups and concentrates, as well as mineral waters where there is “added sugar” or sweeteners [67]. In practice, constraints on policymakers may push them towards emulating solutions favored by international organizations, however, in South Africa’s case this allowed policymakers be act as a frontrunner for other LMICs [20].

## 5. Discussion

The findings of this paper demonstrate that global-level, core policy ideas on what constitutes an unhealthy diet and how it should be addressed were largely accepted and implemented by the South African government. Our policy document analysis honed in on four policy options to reduce sugar intake identified during the initial scoping review, namely; FBDG, marketing and advertising of foodstuffs and non-alcoholic beverages, regulation on labelling and advertising of foodstuffs, and the taxation of sugar in SSBs. Authorities appear to have responded to policy ideas promoted by international agencies, acting in areas such as food labelling, nutrition claims and taxes on SSBs, as these issues gained attention. The marketing and advertising of foodstuffs and non-alcoholic drinks was the only area to have experienced little progression even though excess weight and obesity are rising rapidly in South African children [68]. On the other hand, policymakers in the finance sector have proactively implemented a tax on SSBs [66]. The following four learnings emerged from the policy analysis:

Policy learning 1: Identify local health priorities and use the WHO ‘roadmap’ for multisectoral and cost-effective policy action

The strategic roadmap of policy options set out in the 2004 WHO Global Strategy and WHO Global Action Plan (2013–2020) has provided a helpful pathway that countries such as South Africa could follow and build upon, in accordance with their own epidemiological and policy experience. Evident from the policy analysis is the fact that global directives have found expression in Africa Regional, South African national and Western Cape provincial policy documents. Within key strategy documents related to obesity and NCDs, policy ideas such as applying multisectoral, cost-effective approaches to advance efforts nationally are strongly iterated and implemented. 

A next step for South African policymakers would be to look at the supply chain as a whole and determine the incentives and disincentives for reducing sugar in manufactured foods throughout the supply chain [69]. Hawkes and Watson (2017), who reviewed the supply chain from production to retail for sugar in the European context, have set out a framework of incentives that can be used by South African policymakers as a starting point for recognizing what are effective “leverage points” for policy actions that create change.

Policy learning 2: Strengthen participation in global and regional decision-making structures

Like other countries belonging to the WHO or the FAO, South Africa must pay for its membership to be able to participate within the decision-making structures of these organizations. South Africa also has rights and obligations associated with its membership [70,71]. Various WHO technical reports were identified but not included in this study and these, together with the interaction between South African policymakers on the development of the sugar tax and the WHO, shows the international organization has tried to support national governments to bridge the gap towards acquiring the level of expertise needed to design and administer taxes that meet policy goals effectively [33,34,72]. On a cautionary note, Diane Stone (2012) has pointed out that international organization’s use of “long-term persuasion exercises” involving scientific evidence and advocacy approaches to promote compliance to best practice can be depicted as “indirect coercion” [73].

The South African regulations relating to the labelling of foodstuffs and non-alcoholic drinks provide some evidence of policy copying from the Codex, hinting at the powerful influence of these international structures. The example of copying cited in the findings relates to sugar claims introduced in the South African regulations of 2014 (No. R146) [40]. Sugar claims are important to the Codex guideline to ensure that the consumer is not misled or unduly influenced in their purchasing decision [74]. Research shows that when a claim of “no added sugar” is presented on a product, consumers assume that it is healthier [74]. 

It makes sense that governments take responsibility for the health of their people. However, the responsibility of managing and solving the problem of unhealthy diets and reducing sugar consumption has effectively fallen squarely on the shoulders of countries. This, despite international-level policies’ awareness of the wider influence of global forces on food and supply chains, LMIC’s weak capacity to resist these trends and the relatively absent role of regional authorities on this issue. A United States Department of Agriculture (USDA) report entitled “*Inconsistent Participation of Southern African Countries at Codex*” (2018) found that, with the exception of South Africa (which attends at least three-quarters of the meetings held annually), attendance is otherwise poor among countries in the region, because of a “*lack of financial, human and technical resources, and consequently absence of scientific data to support country positions*” [41]. These meetings are an important platform for countries in the region to consolidate their position on issues that will be discussed at the annual Codex meetings at the global level, and to be able to take a stand against powerful trading partners such as the European Union, whose proposals might impact the interests of Southern African countries [41].

Policy learning 3: Build leadership, national capacity and increase provision for technical assistance to overcome policy constraints

To gauge progress on implementing national policies on NCDs within budget allocations at the country-level, the WHO conducted surveys from 2010 to 2013 [42]. The results of the surveys found that countries were “*struggling to move from commitment to action*” [42]. A variety of reasons for this are documented in the WHO’s Director General report on the UNIATF and are mentioned in the study results. These reasons range from shortcomings in terms of leadership and national capacity, to issues of private interference and a lack of multilateral and bilateral action to support requests made for technical assistance [42]. 

Documents reviewed after the policy analysis made clear that taxation on SSBs in South Africa was constrained and delayed by resistance to the policy from sectors outside of health, such as the labor and food industries and the private sector. On hearing the comments from stakeholders, the National Treasury and the South African Revenue Service (SARS) opted to “*amend the design of the tax to mitigate job losses*” [75]. The documents reviewed for the analysis provide limited insights into the consultation process that took place prior to the introduction of the HPL. However, South African policymakers are fortunate in that they have experience in this regard related to implementing the tobacco tax in the face of strong push-back; those working within the revenue services know “*that any tax proposal is subject to extreme lobbying*” and delays, “*as affected stakeholders will benefit from any delay*” [75]. This type of interference poses a serious threat to LMICs’ ability to institute an effective fiscal measure.

Policy learning 4: Evidenced-based approaches can support advocacy efforts to help combat private sector interference

The WHO recommends a tax percentage of 20% based on the price of the product (called an ad valorem tax) to discourage consumption, but according to Karim et al. (2021) it does not prescribe what type of structure such a tax should take [76]. As such, South Africa conducted an economic evaluation to determine which type of intervention would render the most effective health gains, as described by the WHO’s “gold standard” proposals. Subsequently, a policy brief explored the rationale for different policy designs by examining other countries’ interventions. Using this as inspiration, South Africa employed a tax to meet local requirements and conditions [66]. However, as this analysis highlights, the private sector has been criticized for obstructing policies to reduce sugar intake. The process of introducing the HPL in South Africa overtly depicts what private sector resistance looks like at the country level. In South Africa, the beverage and sugar industries play an important role in the local economy, adding to their political weight. According to Cullinan et al. (2020), these industries, which include global giant Coca-Cola Beverages South Africa, launched a well-organized opposition and apparently exploited the country’s economic vulnerability to “*attack the proposed tax*” arguing that it would cause “*significant job losses*” [77]. A South African policy brief proposed instituting a tax of 20% on the price, but what transpired in practice was a substantially more conservative calculation of 2.1 cents per gram of the sugar content that exceeds 4 g per 100 mL for beverages [66]. The application of 4 g versus the 5 g threshold originally mentioned in the position paper, and which translates to around 12%, was criticized by commentators of the draft bill [14]. 

Locally, evidence was used by various stakeholders, including civil advocates, government, international organizations, and industry, to advocate for their standpoint on the tax [72,75,77]. The Priority Cost-Effective Lessons for System Strengthening in South Africa (PRICELESS), a research-to-policy unit, generates critical local evidence and engages policymakers in government. It collaborated with Healthy Living Alliance (HEALA) to launch an evidence-based advocacy campaign to inform people about the harms of high-sugar intake, and to generate public support for the initiative [77]. These advocacy groups have also had experience with the tobacco industry with the tax on cigarettes. Other LMICs are unlikely to have the advantage of a well-coordinated civil-society response to orchestrate a coherent counterattack on threats to the local implementation of policy ideas on sugar reduction.

At the same time, the South African civil society advocacy group HEALA has made a strong call to implement the WHO recommendations on the marketing of foods and beverages to children and for the country to stand up to the Big Food industry where marketing of unhealthy foods to children is concerned [68]. Implicit in the statement is that the food industry and private sector have potentially been hampering the adoption of global dictates that have been on the table for almost a decade. The South Africa Child Gauge (2020) has also highlighted that “progressive restrictions” on the marketing of unhealthy foods to children in South Africa is justified based on research on advertising to the youth [78]. 

## 6. Relevance for Other LMICs and Further Research

It is our view that much could be learnt from the South African experience for other LMICs related to the successful adoption of the policy measures. The South African government stipulates that changing behavior and the food environment in various settings such as schools, early childhood centers and public spaces *“warrants a radical adoption of interventions that will influence change in these settings*” [79]. This study did not analyze food programs and agriculture policies, although the policy documents were searched for. As such, this area should be explored more fully to provide a comprehensive assessment of government efforts to promote healthy eating [35]. Promising government initiatives aimed in this direction and mentioned in the documents include food gardens in schools, urban agriculture (such as community gardens), home and school gardens, and non-profit garden centers [29,79]. Lastly, learnings from the South African sugar industry’s experience and strategic comeback as outlined in the Sugarcane Value Chain Master Plan released in 2020 could hypothetically offer lessons for other sugarcane and beet-growing countries faced with similar changes [16]. 

## 7. Conclusions

In conclusion, this study provides a comprehensive policy document analysis within the parameters specified and has used the designated conceptual framework to explore policy ideas related to sugar reduction and nutrition led NCDs as they have emerged over the span of 20 years. It has demonstrated that globally held policy ideas about unhealthy diets and limiting sugar intake to prevent and control NCDs have found expression in the South African context. As far as we are aware, no other studies have applied Dolowitz and Marsh’s policy transfer framework (2000) to analyze policy documents concerned with nutrition-related health. Our policy analysis also stands out in this area for its approach to analyzing multilevel policy transfer, and for taking into consideration various policy options pertaining to the reduction in sugar intake for the prevention and control of NCDs—certainly within the South African health policy landscape, if not globally. Therefore, this study offers a unique perspective and departure point for other researchers in HPA in LMICs hoping to understand the transfer of policy ideas on reducing sugar intake for preventing and controlling NCDs within their specific regional contexts. A future avenue for research could involve application of the policy transfer framework to another LMIC for a similar purpose.

## Figures and Tables

**Table 1:** Policy transfer framework and structure of the policy analysis results.

Policy Transfer Framework Questions	Evident from the Policy Analysis
Who is involved in the policy transfer process and why engage in transfer?	International organizations, national government and private sector (food and beverage industry)
Where are policy directives drawn from?	Global recommendations from the WHO/Food and Agricultural Organization (FAO) and Codex Alimentarius (the ‘Codex’)
What elements of policy are transferred?	Policy ideas organized by three themes: the problem frame, suggested approaches (multisectoral/cost-effective) for addressing the problem of unhealthy diets and high sugar intake and policy options
What are the different degrees of transfer?	Mostly policy dictates are emulated nationally, and very occasionally some wording is copied
What restricts or facilitates the policy transfer process?	Private sector interference, skills capacity and policy expertise within government can constrain policy transfer, while international organizations actions help facilitate it

**Table 2:** Evidence of private sector interference.

Source	Textual Quote
Global United Nations Economic and Social Council (ECOSOC). UNIATF on the Prevention and Control of NCDs E/2017/54	“*Private sector interference that blocks governments in their efforts to implement certain very cost-effective and affordable measures to attain target 3.4 of the Sustainable Development Goals (for example, increasing excise taxes and prices on tobacco products, alcoholic beverages and sugar-sweetened beverages)”* [42].
WHO, Montevideo Roadmap 2018–2030 on NCDs as a Sustainable Development Priority, WHA71.2 Annex, 2018	”*One obstacle at country level is the lack of capacity to effectively address public health goals when they are in conflict with private sector interests, in order to effectively leverage the roles and contributions of the diverse range of stakeholders in combatting NCDs*” [48].
Africa Region WHO Regional Office for Africa. Status of Implementation of the Four Time-bound Commitments of NCDs	“*Tackling NCD risk factors in the region is hampered by the interference of the tobacco, alcohol and food industries*” [47].

**Table 3:** A roadmap for action: the WHO policy proposals and where they found expression at the national/subnational level.

Global WHO Global Strategy on Diet, Physical Activity, and Health (2004) Policy Recommendations and Examples for Member States	National South African and Western Cape Corresponding policies Addressing Unhealthy Diets and High Sugar Intake
National strategies, policies and action plans need broad support	
National strategies on diet and physical activity National dietary guidelines	Food-based Dietary Guidelines (FBDG-2013) South African Plan for the Prevention and Control of NCDs (2013–2020) Strategy for the Prevention and Control of Obesity in South Africa (2016)
Governments should provide accurate and balanced information	
Education, communication, and public awareness Adult literacy programs Marketing, advertising, sponsorship, and promotion Labelling health claims	Guideline 14: Marketing and advertising to children Workplace Healthy Eating guidelines (2016) Regulations relating to the labelling and advertising of foodstuffs including (R. 642, 2007); (R. 146, 2010), (R. 429, 2014)
National food and agricultural policies should be consistent with the protection and promotion of public health	
Promotion of food products consistent with a healthy diet Fiscal policies Food programs Agricultural policies	National Development Plan 2030 (2010) Healthcare 2030 (2014) National Food and Nutrition Security Plan 2018–2023 (2017) Household Food and Nutrition Security Strategic Framework (2016) Rates and Monetary Amounts and Amendment of Revenue Laws Bill 26 of 2017 (HPL, 2018)
School policies and programs should support the adoption of healthy diets and physical activity	National School Nutrition Programme (NSPN) Tuck Shop Guidelines (2014) Western Cape school healthy eating guide (2020)

**Table 4:** Textual comparison of the definition of sugar.

Source	Textual Quote
Global 2004 WHO Global Strategy	“*The term ‘free sugars’ refers to all monosaccharides and disaccharides added to foods by the manufacturer, cook or consumer, plus sugars naturally present in honey, syrups and fruit juices”* [35].
National South African FBDG-2013	“*Definition of ‘added sugars’ means any sugar added to foods during processing, and includes but is not limited to: mono and disaccharides (sugars), honey, molasses, sucrose with added molasses, coloured sugar, fruit juice concentrate, deflavoured and/or deionised fruit juice and concentrates thereof, fruit nectar, fruit and vegetable pulp, dried fruit paste, high-fructose corn syrup (HFCS), malt or any other syrup of various origins, whey powder, milk solids or any derivative thereof”* [53].

**Table 5:** Codex wording compared with South African regulations on labelling and advertising of foodstuffs.

Source	Textual quote
Global Codex guidelines for use of nutrition and health claims, CAC/GL 23-1997	***“Non-addition of sugars*** *Claims regarding the non-addition of sugars to a food may be made, provided the following conditions are met: Claims* *(a) No sugars of any type have been added to the food (examples: sucrose, glucose, honey, molasses, corn syrup, etc.);* *(b) The food contains no ingredients that contain sugars as an ingredient (Examples: jams, jellies, sweetened chocolate, sweetened fruit pieces, etc.)”*
National South African regulations relating to the labelling and advertising of foods, 2014 (No. R. 429)	***“Non-addition claims for sugar(s)*** *(iii) Claims regarding the non-addition of sugars to a food may be made, provided the following conditions are met:* *(aa) the food contains no ingredients that contain sugars as part of an ingredient, such as, but not limited to, jams, jellies, sweetened chocolate, sweetened fruit pieces”*

## Data Availability

Data sharing is not applicable to this article. No new data were created or analyzed in this study.

## Supplementary Materials

The following are available online at https://www.mdpi.com/article/10.3390/ijerph191811828/s1, Figure S1: Policy transfer framework, Table S1: Codebook of the policy transfer framework, Table S2: List of included policy documents.
