# Supplementary material for: Reducing Sugar Intake in South Africa: Learnings from A Multilevel Policy Analysis on Diet and Noncommunicable Disease Prevention
Source: Int J Environ Res Public Health. 2022 Sep 19;19(18):11828. doi: 10.3390/ijerph191811828 (PMC9517510; doi:10.3390/ijerph191811828)
Supplement: Supplementary file 1 [file ijerph-19-11828-s001.zip › Figure S1 Policy transfer framework.pdf]

Figure S1: Policy transfer framework adapted from Dolowitz and Marsh (1996 and 2000)

	Why transfer?								
Want to...		Have to..	Who is involved in transfer?	What is transferred?	From where?	Degree of transfer	Constraints on transfer	How to demonstrate policy transfer	How transfer leads to policy failure
Voluntary	Mixtures	Coercive			Multilevel				
Lesson drawing (perfet rational-ity)	Lesson drawing (bounded rationality)	Direct imposition	Elected officials	Policies	Global	Copying	Policy compexity	Reports (Commissioned/ Uncommissioned)	*Uniformed
	International pressures (Image) (Consensus) (Perceptions)		Bureaucrats/Civil servants	Ideologies	Regional	Emulation	Past policies	Conferences	*Incomplete
	Externalities		Consultants/ think tanks/ transnational corporations	Attitudes/cultural values	*National	Mixtures	Structural/ institutional feasibility (ideology) (cultural) (proximity) (technology) (economic) (bureaucratic)	Meetings/visits	*Innappropriate
	Conditionality (Loans) (Conditions attached to business activity)		Supranational institutions	*Programmes	*Subnational	Inspiration	Language	*Better practices/ online sharing platforms	
	Obligations			*Negative lessons				*Frameworks	
Adaptation of the model<br>Explanatory note				Some aspects were not considered in detail (*) due to the focus of the review on policy directives and recommendations	The framework has been simplified. This includes transfer within-in-nation (*)			Mechanisms for sharing policy learnings identified (*) and added while the category for media was omitted	Due to type of policy documents and research question focus this category was not included in the analysis (*)
