# Supplementary material for: Reducing Sugar Intake in South Africa: Learnings from A Multilevel Policy Analysis on Diet and Noncommunicable Disease Prevention
Source: Int J Environ Res Public Health. 2022 Sep 19;19(18):11828. doi: 10.3390/ijerph191811828 (PMC9517510; doi:10.3390/ijerph191811828)
Supplement: Supplementary file 1 [file ijerph-19-11828-s001.zip › Table S1 Codebook of the policy transfer framework.pdf]

Table S1: Codebook of the Policy transfer framework*

CATEGORY	SUB-CATEGORY	DESCRIPTION
**WHY TRANSFER CONTINUUM**		
Why transfer? Is transfer voluntary? A continuum between want to and have to.	Voluntary	Lesson drawing (perfect rationality) commonly arises from dissatisfaction with the status quo as a result of policy failure or improper policy functioning. This leads policymakers or the public to seek "solutions" or lessons without external pressure [1]. Characteristic of seeking out policy lessons under these conditions is having full information about options in choice of strategies best to pursue goals e.g. WHO "best buys".
	Mixture of voluntary and coercive elements	Lesson drawing (bounded rationality) occurs when decision-making is restricted by human (e.g. skills/capacity) or organisational characteristics (e.g. technology). The speed of technology can pressure governments to change and choose alternate institutional structures or practices [1].
		Indirect coercive (externalities or functioning co-dependence) may include international pressure, perceptions by policymakers such as the fear of being left behind or conditions such as loans or obligations.
	Coercive	Direct imposition from pressure groups, political parties, policy entrepreneurs/experts or supranational institutions that chooses to encourage or even forces a government to assume policy lessons following international consensus on an issue [1].
**ACTORS**		
Who is involved in policy transfer?	Elected officials	Political representatives may either be directly or indirectly involved in the policy transfer process through setting a country's value direction or legitimating the adoption of programmes [2].
	Bureaucrats/civil servants	Public servants may be involved in two ways, either through the process of generating research that can support policymakers in making decision-making or by facilitating information flow between higher-up officials [2].
	Pressure groups	Institutions, interest groups or collections of stakeholders in issue networks are internationally well-connected and will advocate on a specific policy agenda [3].
	Policy entrepreneurs/experts	Think tanks, knowledge institutions (KI), academics, consultants and other experts are important for three reasons, namely: the advocacy of lessons or ideas: their ability to collect, sort and distribute specialised information; and, lastly, for having well-developed networks formed as a result of their expertise in subject area [1,4].
	Supra-national governmental and non-governmental institutions	Intergovernmental and international organizations such as the European community, OECD, World Bank, United Nations and trans-national corporations encourage exchange of ideas between countries. Well-connected with government, they also directly or indirectly lobby for certain policies. In addition, global agencies tend to focus on programmes in developing countries and often promote comparison between countries [1].

* The Policy Transfer Framework is taken from Dolowitz and Marsh (1996 and 2000) and has been adapted for the purposes of the study, *Reducing Sugar Intake in South Africa: Learnings from A Multilevel Policy Analysis on Diet and Noncommunicable Disease Prevention*.

**POLICIES**		
What is transferred?	Policy goals	The goals describe the desired outcomes or what should be realised as a result of implementing the policy. Goals may be transferred either in whole or part.
	Content	The language or exact wording may be paraphrased or policy discourse expressed in policies used in other contexts. Initial studies on policy diffusion tended to focus on the transfer of structure, this evolved to include content as it became known as policy transfer [5]. The content includes policy proposals that articulate a set of preferred options or recommendations.
	Policy instruments	Economic tools such as taxes, regulations or productivity measures are the types of policy instruments or administration techniques that may be adopted [5]. The early literature indicated that this would take place from one country to another.
	Policy programmes	A policy programme may be described as specific courses of action or called an intervention. Programmes are implemented to create improvement in response to identified challenges. Policies which are broader intentions of statement can have multiple programmes, whereas a programme is "complete course of action in and of itself" [5]. Policy programmes are usually transferred at the country level, between or within countries.
	Institutions	Institutions including those used to implement policy can be transferred [1].
	Ideologies	The collection of ideas or beliefs of a government or institution underpinning a policy can be transferred [1].
	Ideas, attitudes and concepts	Policy ideas spread through various avenues including professional organisations, networks of specialists, policy entrepreneurs, the media or chance communication [6]. Ideas from outside the political system can persuade politicians to elevate an issue onto the policy agenda [6]. The transfer of belief systems, attitudes political culture underlying policies may be more challenging to identify than examples of structure [6].
	Negative lessons	Negative lessons can be extracted to illustrate what should not be done [1].
**DIRECTION OF TRANSFER**		
Where was it transferred from to where? I.e. From where are lessons drawn?	Multilevel governance	Multi-level governance is becoming more frequent phenomenon, with horizontal linkages established between international, national, regional and local levels [7].
**DEGREE OF TRANSFER**		
How much is transferred?	Copied	A policy is transferred directly and completely. Policy goals, content and programmes are predominantly transferred.
	Emulated	The ideas supporting the policy or programme are transferred [5]. Copying an entire policy or programme is rejected, however there is an acceptance that the policy identified elsewhere provides the best standard for designing legislation locally and then the idea is built up in a local environment [1] [2].
	Mixtures (hybridisation/synthesis)	Combinations involving several different policies [5].
	Inspiration	Policy elsewhere may be the inspiration for policy change, however the final outcome of the policy is not contingent on the original idea [5].

**CONSTRAINTS ON TRANSFER**		
What determines the likelihood and success of policy transfer?	Policy complexity	Complex policies such as those with multiple components are more challenging to transfer, compared to simpler polices where there is a single goal, less complicated problem to solve, more information about the policy, fewer perceived side-effects and more easily predictable outcomes [1].
	Structural Institutional Feasibility	The ideological or cultural similarity between the sites from which policy is being transferred are factors that may influence the feasibility of policy transfer, as are physical proximity, technology in use, or the economic, bureaucratic or political structure of country [1].
**DEMONTRATE TRANSFER**		
How was the policy transfer marketed to stakeholders?	Reports	Commissioned or un-commissioned reports are principal information sources used to articulate the proposed policy change. Reports can provide details on traits of the policy considered that could have relevance when transferring policy to another setting [2].
	Internet/online platforms	Much communication these days takes place online and these technologies also play a key role in policy formation. Ideas can be easily shared and feedback obtained from interested parties [2].
	Better practices*	Best practices of policies and programmes have become common use to influence the process of policymaking and is considered to be an effective means of promoting policy transfer. A perception of better practices can be that they may be applied with similar effect in another setting and that their development and dissemination will result in positive outcomes of policy and practice elsewhere [8].
	Conferences/meetings/visits	Conferences, roundtable events, group meetings or visits may be arranged with certain target audiences who have a stake in the policy to communicate the proposed change with them and also to get their feedback on the proposal [2].

References

1. Dolowitz, D.; Marsh, D. Who Learns What from Whom: A Review of the Policy Transfer Literature. *Polit. Studies* **1996**, *44*, 343-357, doi:10.1111/j.1467-9248.1996.tb00334.x.
2. Azatyan, O. Policy Transfer As Alternative to Policy Making Locally in Armenia. **2017**.
3. Richardson, J. Government, interest groups and policy change. *Political studies* **2000**, *48*, 1006-1025.
4. Stone, D. Non-governmental policy transfer: the strategies of independent policy institutes. *Governance* **2000**, *13*, 45-70.
5. Dolowitz, D.P.; Marsh, D. Learning from Abroad: The Role of Policy Transfer in Contemporary Policy-Making. *Governance* **2000**, *13*, 5-23, doi:10.1111/0952-1895.00121.
6. Wolman, H. Director, George Washington Institute of Public Policy Revised October 6, 2009. In Proceedings of the Presentation to Gastein European Forum for Health Policy, 2009.
7. Evans, M.; Davies, J. Understanding policy transfer: A Multi-level, multi-disciplinary perspective. *Public administration* **1999**, *77*, 361-385.
8. Stead, D. Best practices and policy transfer in spatial planning. *Planning Practice and Research* **2012**, *27*, 103-116.
