# Supplementary material for: Reducing Sugar Intake in South Africa: Learnings from A Multilevel Policy Analysis on Diet and Noncommunicable Disease Prevention
Source: Int J Environ Res Public Health. 2022 Sep 19;19(18):11828. doi: 10.3390/ijerph191811828 (PMC9517510; doi:10.3390/ijerph191811828)
Supplement: Supplementary file 1 [file ijerph-19-11828-s001.zip › Table S2 List of included policy documents.pdf]

Table S2: List of included policy documents*

Document type	Reference	Year	Organization	Title
**Global**				
Report	[1]	2004	COAG	FAO's Proposed Follow-up to the Report of the Joint WHO/FAO Expert Consultation on Diet, Nutrition and the Prevention of Chronic Diseases
Resolution and decisions, Annexes	[2]	2004	WHO	Fifty-Seventh World Health Assembly. WHA57.17 Global strategy on diet, physical activity and health
Resolution and decisions, Annexes	[3]	2007	WHO	Sixtieth session, First Special session, WHA60.23 Prevention and control of noncommunicable diseases: implementation of the global strategy
Resolution and decisions, Annexes	[3]	2007	WHO	Sixtieth session, First Special session, WHA60.24 Health Promotion in a Globalized world
Report on matters arising from WHO/FAO	[4]	2007	CODEX	Codex committee on food labelling, thirty-fifth session, Ottawa, Canada, April 30 - May 4 2007
Guidelines and standards	[5]	2007	CODEX	Codex Alimentarius Food Labelling.
Resolution and decisions, Annexes	[6]	2008	WHO	Sixty-First World Health Assembly WHA61/2008/REC/1, WHA61.14 Prevention and control of noncommunicable diseases: implementation of the global strategy
Resolution and decisions	[7]	2010	WHO	Sixty-third World Health Assembly: Resolutions and decisions annexes. WHA63.14 Marketing of food and non-alcoholic beverages to children
Declaration	[8]	2011	WHO	First Global Ministerial Conference on Healthy Lifestyles and Noncommunicable Disease Control Moscow, 28-29 April 2011. Moscow Preamble
Report of the Secretary General	[9]	2011	UNGA	Prevention and control of non-communicable diseases Report of the Secretary-General
Resolution	[10]	2012	UNGA	Resolution adopted by the General Assembly: Political Declaration of the High-level Meeting of the General Assembly on the Prevention and Control of Non-communicable Diseases, 66th session
Matters arising from WHO/FAO	[11]	2012	CODEX	Joint FAO/WHO Food Standards Programme Committee on Food Labelling Fortieth Session. Ottawa.
Report of the Director-General	[12]	2012	UNGA	Note by the Secretary-General transmitting the report of the Director-General of the World Health Organization on options for strengthening and facilitating multisectoral action for the prevention and control of non-communicable

* The list of included articles is for the study entitled: *Reducing Sugar Intake in South Africa: Learnings from A Multilevel Policy Analysis on Diet and Noncommunicable Disease Prevention*.

Resolution and decisions, Annexes	[13]	2012	WHO	Sixty-Fifth World Health Assembly WHA65/2012/REC/1., WHA65(8) Prevention and control of noncommunicable diseases: follow-up to the High-level Meeting of the General Assembly on the Prevention and Control of Non-communicable Diseases
Guidelines	[14]	2013	CODEX	Guidelines for the Use of Nutrition and Health Claims (CAC/GL 23-1997)
Report of the Director-General		2013	UNGA	Prevention and control of NCDs, A68/650
Resolution and decisions, Annexes	[15]	2013	WHO	Sixty-Sixth World Health Assembly WHA66/2013/REC/1, WHA66.8 Follow-up to the Political Declaration of the High-level Meeting of the General Assembly on the Prevention and Control of NCDs
Report of the Director-General	[16]	2013	WHO	Note by the Secretary-General transmitting the report of the Director-General of the World Health Organization on the prevention and control of non-communicable diseases A/68/650.
Declaration	[17]	2014	FAO	Conference Outcome Document: Rome Declaration on Nutrition. Second International Conference on Nutrition
Strategies and plans	[18]	2014	FAO	Conference Outcome Document: Framework. Second International Conference on Nutrition.
Guideline	[19]	2015	WHO	Guideline: Sugars intake for adults and children
Report of the Director General	[20]	2017	UN Economic and Social Council (ECOSOC)	United Nations Inter-Agency Task Force on the Prevention and Control of Non-communicable Diseases E/2017/54.
Resolution and decisions, Annexes	[21]	2017	WHO	Seventieth World Health Assembly WHA70/2017/REC/1, WHA70(19) Report of the Commission on Ending Childhood Obesity
Guidelines and standards	[22]	2017	CODEX	Codex Guidelines on Nutrition Labelling Revised
Resolution and decisions, Annexes	[23]	2018	WHO	Seventy-First World Health Assembly WHA71/2018/REC/1, WHA71.2 Preparation for the third High-level Meeting of the General Assembly on the Prevention and Control of Non-communicable Diseases, to be held in 2018
Resolution	[24]	2018	UNGA	Political declaration of the third high-level meeting of the General Assembly on the prevention and control of non-communicable diseases A/RES/73/2
Report of the Director General	[25]	2019	UN Economic and Social Council (ECOSOC)	United Nations Inter-Agency Task Force on the Prevention and Control of Non-communicable Diseases E/2017/54.
Report of the Director General	[26]	2019	WHO	Outcome of the Second International Conference on Nutrition: Report by the Director-General A72/58.
Guidelines	[27]	2020	South African Revenue Services	Excise External Policy. Health Promotion Levy on Sugary Beverages
**Africa region**				
Strategies and plans	[28]	2012	WHO	Oral health in the WHO African Region: A regional strategy (1999–2008) in Compendium of Health Strategies
Strategies and plans	[29]	2016	WHO	Regional Oral Health Strategy 2016-2025: Addressing Oral diseases as Part of Non-communicable Diseases

Report of the Secretariat	[30]	2018	WHO	Status of the Implementation of the Four Time-bound Commitments of NCDs
**South Africa**				
Guidelines	[31]	2007	Department of Health	Draft Guidelines relating to regulations governing the labelling and advertising of foods
Regulations	[32]	2010	Department of Health	Foodstuffs, Cosmetics and Disinfectants Act, 1972: Regulations relating to the labelling and advertising of Foods
Strategies and plans	[33]	2010	National Planning Commission	Our future – make it work. National Development Plan 2030
Declaration	[34]	2011	Department of Health	South African Declaration on the Prevention and Control of NCDs
Strategies and plans	[35]	2012	Department of Health	Strategic plan for the prevention and control of non-communicable diseases 2013–17.
Guidelines	[36]	2013	Department of Health	Food-Based Dietary Guidelines for South Africa.
Guideline and standards	[37]	2014	Department of Basic Education	Food Specifications – for products marketed to the National School Nutrition Programme
Guidelines	[38]	2014	Department of Basic Education	National School Nutrition Programme Guidelines for Tuck Shop Operators.
Regulations	[39]	2014	Department of Health	Foodstuffs, Cosmetics and Disinfectants Act, 1972 (Act 54 of 1972): Regulations relating to the labelling and advertising of foodstuffs: amendment.
Guide	[40]	2016	Department of Health	National guide for healthy meal provisioning in the workplace.
Strategies and plans	[41]	2016	Department of Health	Strategy for the prevention and control of obesity in South Africa 2015–2020
Strategies and plans	[42]	2017	National Department of Planning	National Food and Nutrition Security Plan 2018-2023
Act	[43]	2017	National Treasury	Rates and Monetary Amounts and Amendment of Revenue Laws Bill 26 of 2017
Strategies and plans	[44]	2020	Department of Trade and Industry	South African Sugarcane Value Chain Master Plan to 2030
Guideline and standards	[45]	2020	Revenue Services (SARS)	Excise External Policy Health Promotion Levy on Sugary Beverages
**Western Cape**				
Strategies and plans	[46]	2014	Western Cape Government Health	Healthcare 2030.
Strategies and plans	[47]	2016	Western Cape Government Health	Household Food and Nutrition Security Strategic Framework.
Guide	[48]	2020	Western Cape Government Education	Healthy Eating Guide

References

1. Food and Agriculture Organization. FAO's Proposed Follow-up to the Report of the Joint WHO/FAO Expert Consultation on Diet, Nutrition and the Prevention of Chronic Diseases COAG/2004/3. 2004.
2. World Health Organization. Fifty-Seventh World Health Assembly WHA57/2004/REC/1. 2004.
3. World Health Organization. World Health Assembly First Special Session. Geneva2007.
4. Codex Alimentarius Commission. Joint FAO/WHO Food Standards Programme Codex Committee on Food Labelly Thirth-Fifth Session CX/FL 07/35/3. 2007.
5. World Health Organization and Food and Agriculture Organization. Codex Alimentarius Food Labelling. 2007.
6. World Health Organization. Sixyt-First World Health Assembly WHA61/2008/REC/1. Geneva2008.
7. World Health Organization. Sixty-Third World Health Assembly WHA63/2010/REC/1. Geneva2010.
8. World Heath Oganization. First Global Ministerial Conference on Healthy Lifestyles and Noncommunicable Disease Control. World Health Organization; 2011.
9. United Nations General Assembly. Prevention and control of non-communicable disease: Report of the Secretary–General. 2011.
10. United Nations General Assembly. Political Declaration of the High-level Meeting of the General Assembly on the Prevention and Control of Non-communicable Diseases A/RES/66/2. 2012.
11. Codex Alimentarius Commission. Joint FAO/WHO Food Standards Programme Committee on Food Labelling Fortieth Session. Ottawa2012.
12. United Nations General Assembly. Note by the Secretary-General transmitting the report of the Director-General of the World Health Organization on options for strengthening and facilitating multisectoral action for the prevention and control of non-communicable diseases through effective partnership. 2012.
13. World Health Organization. Sixty-Fifth World Health Assembly WHA65/2012/REC/1. Geneva2012.
14. Codex Alimentarius Commission. Guidelines for the Use of Nutrition and Health Claims (CAC/GL 23-1997). 2013.
15. World Health Organization. Sixty-Sixth World Health Assembly WHA66/2013/REC/1. Geneva2013.
16. United Nations General Assembly. Note by the Secretary-General transmitting the report of the Director-General of the World Health Organization on the prevention and control of non-communicable diseases A/68/650. 2013.
17. Food and Agriculture Organization and World Health Organization. Conference Outcome Document: Framework. Second International Conference on Nutrition. Rome2014.
18. Food and Agriculture Organization of the United Nations (FAO) and World Health Organization (WHO), editor Conference outcome document: Rome declaration on nutrition. Second international conference on nutrition Rome: FAO and WHO; 2014.
19. World Health Organization. Guideline: Sugar intake for adults and children. 2015.
20. United Nations Economic and Social Council. United Nations Inter-Agency Task Force on the Prevention and Control of Non-communicable Diseases E/2017/54. 2017.
21. World Health Organization. Seventieth World Health Assembly WHA70/2017/REC/1. Geneva2017.
22. Codex Alimentarius Commission. Guidelines on Nutrition Labeling CAC/GL 2-1985. 2017.
23. World Health Organization. Seventy-First World Health Assembly WHA71/2018/REC/1. Geneva2018.
24. Political declaration of the high-level meeting of the general assembly on the prevention and control of non-communicable diseases. A/RES/73/2, (2018).
25. United Nations Economic and Social Council. United Nations Inter-Agency Task Force on the Prevention and Control of Non-communicable Diseases E/2019/55. 2019.
26. World Health Organization. Outcome of the Second International Conference on Nutrition: Report by the Director-General A72/58. Geneva2019.
27. South African Revenue Services (SARS). Excise External Policy Health Promotion Levy on Sugary Beverages. 2020.
28. World Health Organization Africa. Compendium of Public Health Strategies 2012.
29. World Health Organization Africa. Regional Oral Health Strategy 2016-2025: Addressing Oral diseases as Part of Non-communicable Diseases. In: Africa RCf, editor. Ethiopia2016.
30. World Health Organization Regional Office for Africa. Status of Implementation of the Four Time-bound Commitments of NCDs. Republic of Senegal; 2018.
31. South Africa Department of Health. Draft guidelines regulations governing the labelling and advertising of foodstuffs, R642 of 20 July 2007. 2007.
32. South Africa Department of Health. Foodstuffs, Cosmetics and Disinfectants Act, 1972 (Act 54 of 1972) regulations relating to the labelling and advertising of foodstuffs (R. 146). 2010.
33. National Planning Commission. Our future – make it work. National Development Plan 2030. 2010.
34. South Africa Department of Health. South African Declaration on the prevention and control of diseases. 2011.
35. South Africa Department of Health. Strategic plan for the prevention and control of non-communicable diseases 2013–17. 2012.
36. Vorster HH BJ, Venter CS;. Food-Based Dietary Guidelines for South Africa. South African Journal of Clinical Nutrition. 2013;26(3).
37. South Africa Department of Basic Education. Food Specifications – for products marketed to the National School Nutrition Programme 2014.

38. South Africa Department of Basic Education. National School Nutrition Programme Guidelines for Tuck Shop Operators. 2014.
39. South Africa Department of Health. Foodstuffs, Cosmetics and Disinfectants Act, 1972 (Act 54 of 1972) regulations relating to the labelling and advertising of foodstuffs: amendment. 2014.
40. South Africa Department of Health. National guide for healthy meal provisioning in the workplace. Pretoria; 2016.
41. South Africa Department of Health. Strategy for the prevention and control of obesity in South Africa 2015–2020. 2016.
42. South Africa Coordination Committee and Department of Planning; Monitoring and Evaluation (DPME). National Food and Nutrition Security Plan 2018-2023. 2017.
43. South African National Treasury and Revenue Services (SARS). Final Response Document on the 2017 Rates and Monetary Amounts and Amendment fo Revenue Laws Bill – Health Promotion Levy, 2017. 2017.
44. South Africa Department of Trade and Industry. South African Sugarcane Value Chain Master Plan to 2030. 2020.
45. South African Revenue Services (SARS). Excise External Policy Health Promotion Levy on Sugary Beverages. 2020.
46. Western Cape Government. Healthcare 2030. 2014.
47. Western Cape Government. Household Food and Nutrition Security Strategic Framework. 2016.
48. Western Cape Government Education. Healthy Eating Guide. 2020.
